# A cancer-associated fibroblast gene signature predicts prognosis and therapy response in patients with pancreatic cancer

**DOI:** 10.3389/fonc.2022.1052132

**Published:** 2022-11-18

**Authors:** Jinbao Zhang, Meiling Chen, Chuanfa Fang, Peng Luo

**Affiliations:** ^1^ Department of General Surgery, The First Affiliated Hospital of Dalian Medical University, Dalian, China; ^2^ Fujian Provincial Key Laboratory on Hematology, Fujian Institute of Hematology, Fujian Medical University Union Hospital, Fuzhou, China; ^3^ Department of Gastroenteric Hernia Surgery, Ganzhou Hospital Affiliated to Nanchang University, Jiangxi, Ganzhou, China

**Keywords:** pancreatic cancer, cancer-associated fibroblast, prognostic, survival, biomarker

## Abstract

Pancreatic cancer is a lethal malignancy with a 5-year survival rate of about 10% in the United States, and it is becoming an increasingly prominent cause of cancer death. Among pancreatic cancer patients, pancreatic ductal adenocarcinoma (PDAC) accounts for more than 90% of all cases and has a very poor prognosis with an average survival of only 1 year in about 18% of all tumor stages. In the past years, there has been an increasing interest in cancer-associated fibroblasts (CAFs) and their roles in PDAC. Recent data reveals that CAFs in PDAC are heterogeneous and various CAF subtypes have been demonstrated to promote tumor development while others hinder cancer proliferation. Furthermore, CAFs and other stromal populations can be potentially used as novel prognostic markers in cancer. In the present study, in order to evaluate the prognostic value of CAFs in PDAC, CAF infiltration rate was evaluated in 4 PDAC datasets of TCGA, GEO, and ArrayExpress databases and differentially expressed genes (DEGs) between CAF-high and CAF-low patients were identified. Subsequently, a CAF-based gene expression signature was developed and studied for its association with overall survival (OS). Additionally, functional enrichment analysis, somatic alteration analysis, and prognostic risk model construction was conducted on the identified DEGs. Finally, oncoPredict algorithm was implemented to assess drug sensitivity prediction between high- and low-risk cohorts. Our results revealed that CAF risk-high patients have a worse survival rate and increased CAF infiltration is a poor prognostic indicator in pancreatic cancer. Functional enrichment analysis also revealed that “extracellular matrix organization” and “vasculature development” were the top enriched pathways among the identified DEGs. We also developed a panel of 12 genes, which in additional to its prognostic value, could predict higher chemotherapy resistance rate. This CAF-based panel can be potentially utilized alone or in conjunction with other clinical parameters to make early predictions and prognosticate responsiveness to treatment in PDAC patients. Indeed, it is necessary to conduct extensive prospective investigations to confirm the clinical utility of these findings.

## Introduction

Pancreatic cancer is the third main cause of cancer-related mortality in both male and females (1). Only in 2022, an approximate of 62210 cases are estimated to be diagnosed in the United States with this cancer of which 49830 are anticipated to die (1). Smoking (2), obesity (3), diabetes (4), alcohol consumption (5), and *Helicobacter pylori* infection (6) are the major risk factors linked to this malignancy. Pancreatic ductal adenocarcinoma (PDAC) and pancreatic neuroendocrine neoplasm (PanNEN) are the two primary histological subtypes of pancreatic cancer of which the former accounts for 90% of all pancreatic cancer cases, whereas PanNEN accounts for only 3–5% of all cases (7). Surgical resection, chemotherapy, and radiotherapy are the major conventional therapeutic approaches; however, surgical excision is the only current therapy that can be potentially curative in comparison to other clinical approaches (8). Nonetheless, patients diagnosed with PDAC, show poor survival rates mainly due to their advanced stage at diagnosis, local relapse, and distant metastasis (9). Thus, obtaining adequate knowledge on the cellular and molecular alterations associated with therapeutic response and clinical prognosis is a prerequisite to an efficient treatment for this disease.

The “ecological niche” of cancer cells, the so-called tumor microenvironment (TME), is believed to be among the major drivers of tumor growth, metastasis, and drug resistance (10–12). The TME is comprised of various cellular components like fibroblasts, endothelial cells, immune cells, adipocytes and neuroendocrine cells as well as extracellular elements such as extracellular matrix (ECM) and tumor-stimulating molecules (e.g., cytokines, chemokines, etc.) (13). Among the different cell populations within the TME, cancer-associated fibroblasts (CAFs) have gained more interest due to their multiple pivotal roles in cancer progression, invasion and metastasis, crosstalk with other immune cells, and ECM remodeling (14). Such conspicuous characteristics have turned CAFs into promising sources of prognostic biomarkers as well as attractive candidates for targeted therapy (15). In this regard, the molecular alterations associated with CAFs have been suggested to reflect an informative image of the tumor status, growth and response to therapy and may therefore, be potentially used for optimizing clinical decisions as well as finding novel diagnostic and prognostic biomarkers (16–18). For instance, Ono et al. discovered that the increased expression of CAF-podoplanin in patients with stage I lung squamous cell carcinoma is a poor prognostic predictor. In another study, Takai et al. showed that targeting CAFs *via* Pirfenidone, could decrease cell viability and collagen production of triple-negative breast cancer cells (19, 20). Such findings emphasize the important role of CAFs as cancer-promoting entities as well as sources for the discovery of biomarkers that could prognosticate the clinical outcome. However, the prognostic and predictive value of CAF-associated biomarkers has not been investigated in PDAC patients, so far. Herein, bioinformatics methodologies are increasingly applied to discover associations between such early molecular-level changes and clinical manifestations. Using high-throughput sequencing data, the underlying pathological mechanisms of heterogeneous diseases like cancer may be uncovered and turned into a more informative measure by comparing the expression networks of various genes in different disease status and/or groups of patients.

In the present study, we employed the data on differentially expressed genes (DEGs) and CAF infiltration from three different databases including Gene Expression Omnibus (GEO), Cancer Genome Atlas (TCGA), and ArrayExpress to study the prognostic potential of CAF-associated signatures in PDAC patients. Subsequently, differential gene expression analysis was performed to elucidate the CAF-associated hub-genes and construct the stromal/CAF risk score through CAF-associated gene profile and CAF infiltration. We also conducted pathway enrichment analysis by Gene Ontology (GO) and Kyoto Encyclopedia of Genes and Genomes (KEGG) databases in order to identify the main molecular pathways associated with the CAF-related DEGs. The correlation between the identified gene profile and overall survival (OS) as well as responsiveness to chemotherapeutic agents was evaluated using univariate COX regression analysis.

## Material and methods

### Data collection and preprocessing

The gene expression data and clinical information of PDAC patients were downloaded from the TCGA database (https://tcga-data.nci.nih.gov/tcga/) using the UCSC Xena portal (https://xenabrowser.net/hub/). The RSEM normalization and log2(x+1) transformation were implemented to reach gene-level transcription values. GEO2R package was used to download normalized expression profiles and the clinical data of GSE57495 and GSE57495 from the GEO database. Ultimately, microarray gene expression data for ArrayExpress was acquired through the accession number E-MTAB-6134. Notably, the mean expression was calculated if a single gene had multiple probes. Genes without expression levels were removed from the analysis. The details of clinical characteristics are presented in **Table 1**. A schematic illustration of the study design has been provided in **Supplementary Figure 1**.

**Table 1:** Clinical characteristics of the cohorts included in this study.

	TCGA	GSE57495	GSE78229	E.MTAB.6134
Sex				
Male	96 (73.1%)	33 (52.4%)	not available	166 (57.6%)
Female	80 (26.9%)	30 (47.6%)	not available	122 (42.4%)
Age				
Median	65	68	not available	not available
Range	35-88	24-86	not available	not available
TNM Stage				
I	21	13	4	12
II	145	50	45	39
III	4	–	–	273
IV	4	–	–	–
not available	2	–	–	–
Grade				
G1	30	not available	2	110
G2	94	not available	24	130
G3	48	not available	21	48
G4	2	not available	1	–
Gx	2	not available	1	–
Follow up				
Alive	84	21	14	107
Death	92	42	35	182
Median OS	15.4	21.1	14.2	20.8

### Assessment of CAF infiltration

Microenvironment Cell Populations-counter (MCP-counter) algorithm supplied by the “immunedeconv” package (https://github.com/omnideconv/immunedeconv) was used to estimate the CAF infiltration score of the patients from the collected datasets. MCP-counter, developed by Becht et al., is a bioinformatics tool to quantify tumor-infiltrating fibroblasts, endothelial cells, and immune cells that relies on a strict and reliable collection of marker genes in solid tumors (21).

### Differentially expressed gene analysis

The samples were divided into two groups based on the CAF infiltration scores calculated by the aforementioned algorithm. After determining the CAF infiltration score, the patients were dichotomized as per the calculated score and labeled as “high” and “low”. Subsequently, the “limma” package of R software was used to identify the DEGs of normalized gene expression data between patients with high and low CAF scores. The overlapping DEGs among the four datasets were identified by the Venn diagram, illustrated by Venndiagram package of R. Expression changes with |LogFC| > 0.5 and adjusted p < 0.05 were deemed significant.

### CAF-based prognostic model construction and validation

The TCGA-PAAD cohort was selected to construct and train a CAF risk model while other cohorts from the GSE57495, GSE78229, and E-MTAB-6134 datasets were used for validation. We evaluated potential genes in two steps to construct a predictive risk score model: first we conducted univariate cox analysis using the “glmnet” package and 12 genes were identified with a p value < 0.05 in four datasets. Secondly, Cox proportional hazards model was constructed. Finally, the “survminer” package of R was utilized to evaluate survival analysis. Kaplan-Meier survival analysis was performed between the low- and high-risk groups.

### Somatic alteration analysis

The mutational profile of the TCGA-PAAD patients were obtained using the UCSC Xena portal. The maftools R package was used to identify and display the top 20 highest mutational frequencies in the both low- and high-CAF-risk cohorts. The Chi-squared analysis was implemented to test the associations between clinical data and the altered genes.

### Functional enrichment analysis

The Hallmark gene sets from MSigDB were utilized for GSEA, which was carried out using the clusterProfiler package, while single sample GSEA (ssGSEA) was carried out using the GSVA method. The implemented cutoff criterion for the gene sets was FDR < 0.05.

### Chemotherapy response prediction

Aiming at improving personalized treatment, the oncoPredict package of R was used to predict drug sensitivity against -5-FU, gemcitabine, and Oxaliplatin. Accordingly, drug sensitivity values (measured by ACU, the area under the concentration–response curve) were estimated followed by a comparison of the values between the high- and low-risk groups. High AUC means low sensitivity.

## Results

### Higher CAF infiltration is associated with poor survival in PDAC patients

**Figure 1:**
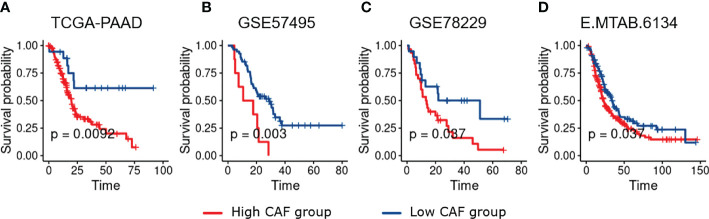
Kaplan–Meier plots of PDAC patients dichotomized based on the infiltration CAF scores calculated using MCP_Counter. Based on the survival analysis, higher CAF Infiltration is associated with worse overall survival in PDAC patients. **(A–D)**, Represent TCGA-PAAD, GSE57495, GSE78229, and E-MTAB-6134 respectively.

Microarray data analysis have been shown to provide gene expression signatures of activated fibroblasts (22). Such signatures have been used to identify particular CAF characteristics in different malignancies including breast cancer, lung cancer, etc (23). Studies in breast cancer have revealed that fibroblast characteristics may, at least partially, impact therapeutic responses (24). As a result, several methodologies have been used to show promising solid evidence, suggesting that CAFs might be utilized for prognostication. Thus, we aimed to investigate whether TME infiltration of CAFs could serve as a prognostic indicator for pancreatic cancer patients. Accordingly, by using MCP-counter, the infiltration CAF scores were estimated in all the studied datasets of PDAC patients. In this context, Kaplan–Meier plots demonstrated that higher CAF infiltration scores were highly associated with poor OS of PDAC patients in all the studied datasets (**Figure 1** **)**.

### Identification of differentially expressed genes

**Figure 2:**
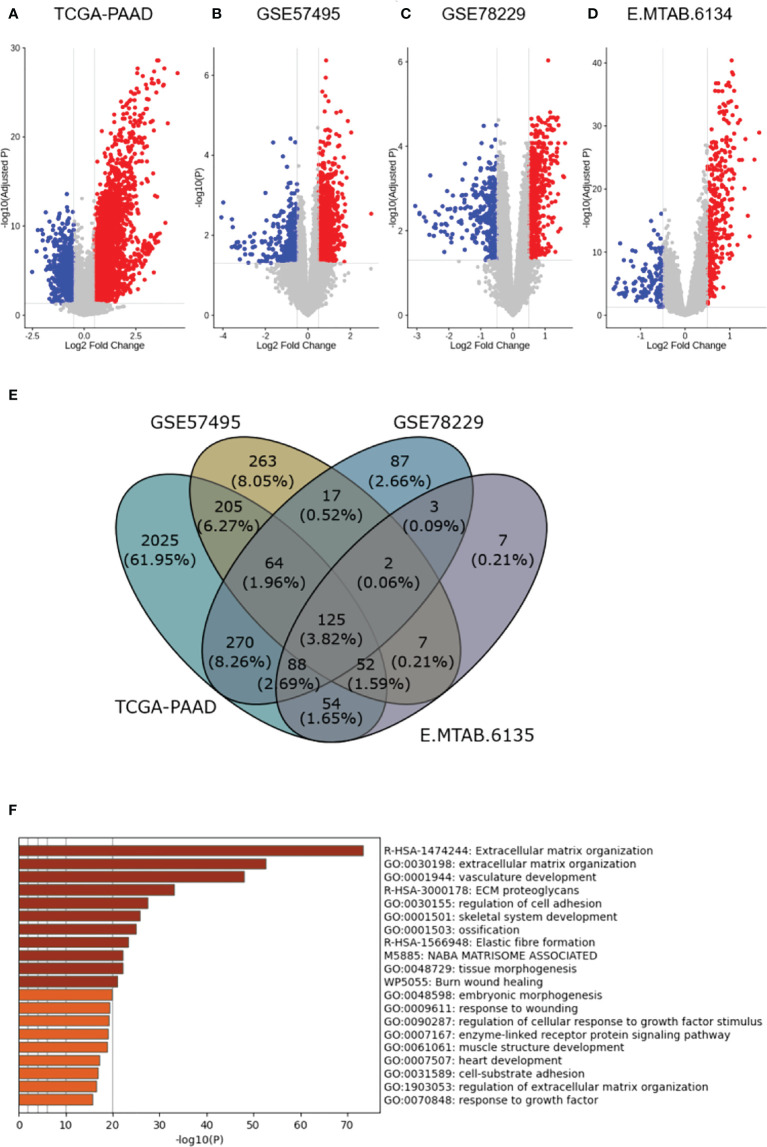
Differential expression analysis. **(A–D)**, Volcano plots representing differential expression analysis results among the high- and low-risk PDAC patients in the studied datasets (TCGA-PAAD, GSE57495, GSE78229, and E-MTAB-6134 respectively). **(E)**, Venn diagram shows 125 differentially-expressed genes overlapping between all four datasets. **(F)**, Overrepresented gene analysis reveals a gene set involved in “Extracellular matrix reorganization” to have the highest relevance to the identified dysregulated genes.

To obtain DEGs between the high- and low-risk PDAC patients, we analyzed all datasets (TCGA-PAAD, GSE57495, GSE78229, and E-MTAB-6134) through the Limma package of R. Adjusted p. value of less than 0.05 and LogFC > 0.5 were the cutoff criteria for the TCGA-PAAD, GSE78229, and E-MTAB-6134 datasets while a raw p value of less than 0.05 was set for the GSE57495 due to the smaller number of candidates after p value adjustment (**Figures 2A–D**). Following the aforementioned criteria, we identified 2883, 735, 656 and 338 upregulated genes in TCGA-PAAD, GSE57495, GSE78229, and E-MTAB-6134, respectively. Ultimately, as shown in **Figure 2E**, 125 overlapping genes among all four datasets were selected for further overrepresented gene analysis. A gene set involved in “Extracellular matrix reorganization” was found to have the highest relevance to the identified dysregulated genes (**Figure 2F**).

### The CAF-based prognostic signature is associated with OS in PDAC patients

**Figure 3:**
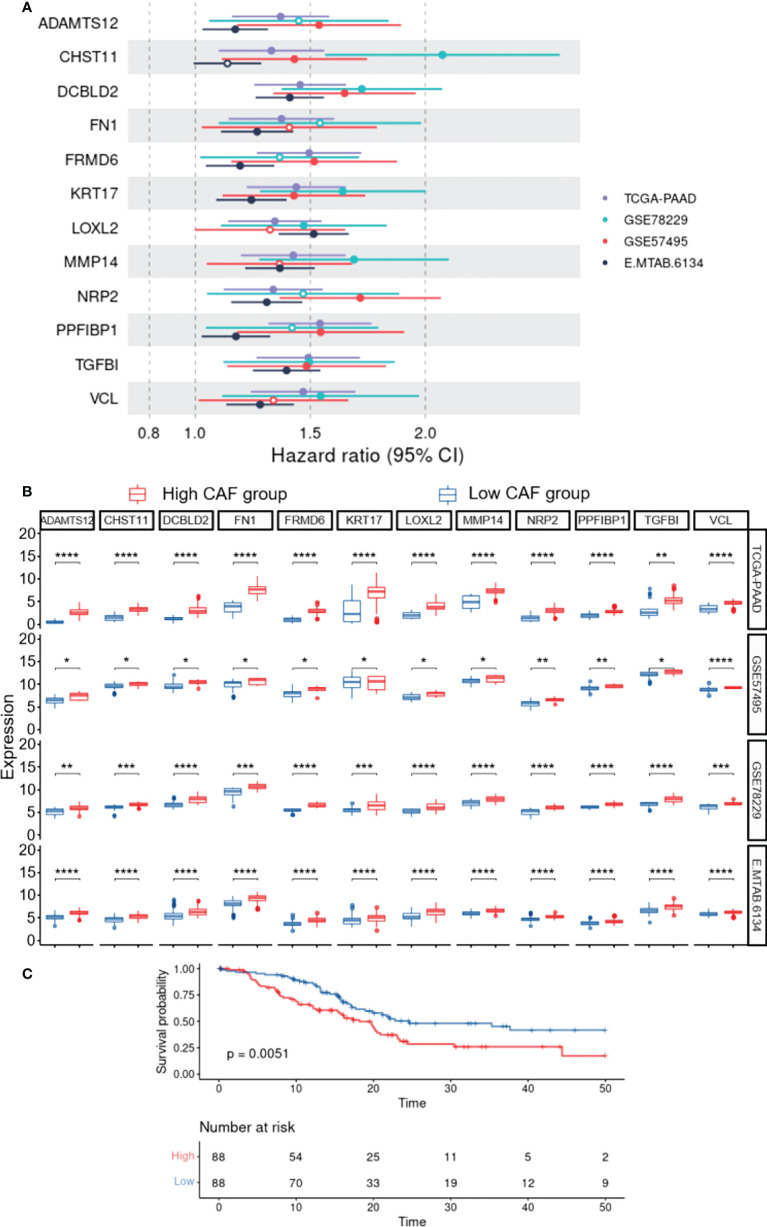
Construction of the CAF-based prognostic risk model. **(A)**, Forest plot representing Hazard ratio of each single gene used to construct the risk model. **(B)**, The expressions between worse and better survival groups across four datasets. **(C)**, Survival analysis reveals the association of the constructed risk model with overall survival in the TCGA-PAAD dataset (P=0.0051). * 0.01 < p-value < 0.05; ** 0.001 < p-value ≤ 0.01; *** 0.0001 < p-value ≤ 0.001; **** p-value ≤ 0.0001.

**Figure 4:**
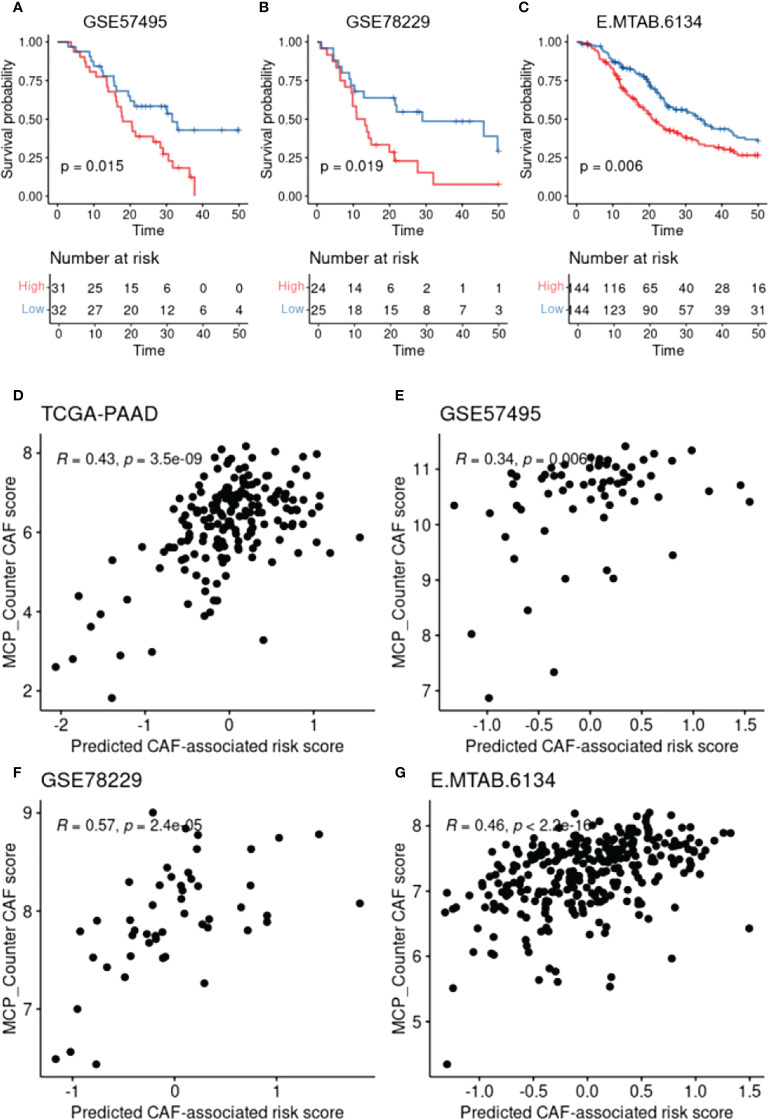
CAF-based prognostic model validation and the association with CAF infiltration. **(A-C)**, Kaplan–Meier plots representing the association of the constructed risk model with overall survival in the validation datasets. **(D-G)**, Scatter plots representing the correlation between the risk model and the calculated CAF infiltration using MCP-Counter.

To construct a CAF-based prognostic risk model, the TCGA-PAAD cohort was selected as the training cohort and the remaining three datasets were used for validation. By conducting univariate Cox regression analysis on the 331 identified up-regulated genes in the training dataset, 12 genes correlating with OS in all four datasets including *ADAMTS12*, *CHST11*, *DCBLD2, FN1, FRMD6, KRT17, LOXL2, MMP14, NRP2, PPFIBP1, TGFB1*, and *VCL* were filtered out (**Figure 3A**). A cox model was constructed based TCGA-PAAD dataset with the following formula: 0.172 * ADAMTS12 + (-0.0870) * CHST11 + 0.127 * DCBLD2 + -0.202 * FN1 + 0.0581 * FRMD6 + 0.266 * KRT17 + (-0.089) * LOXL2 + (-0.196) * MMP14 + (-0.0935) * NRP2 + 0.238 * PPFIBP1 + 0.287 * TGFBI + 0.220 * VCL. The patients were then stratified into high- and low-risk groups based on the median risk score. The expressions between worse and better survival group across four datasets are shown in **Figure 3B**. The CAF-based prognostic risk model was significantly correlated with OS in the TCGA-PAAD dataset (**Figure 3C**). The constructed risk model was also found to be significantly correlated with OS in other validation datasets (**Figures 4A–C**). Further correlation analysis revealed that our risk score is significantly associated with the CAF score calculated by MCP_Counter (**Figures 4D–G**). In addition, Area under the curve (AUC) analysis showed our risk model had AUC values of 0.744, 0.724, 0.761, and 0.617 in the TCGA, GSE57595, GSE78229, and E-MTAB-6134 datasets for predicting 5 years survival (**Supplementary Table 1**), respectively. Based on the decision curve analysis (DCA) analysis, we found that our model showed a higher net benefit in TCGA, E-MTAB-6134 and GSE57495 cohorts in terms of 3- and 5-year survivals.

### The CAF-based signature is associated with certain gene mutations and clinical characteristics in PDAC patients

**Figure 5:**
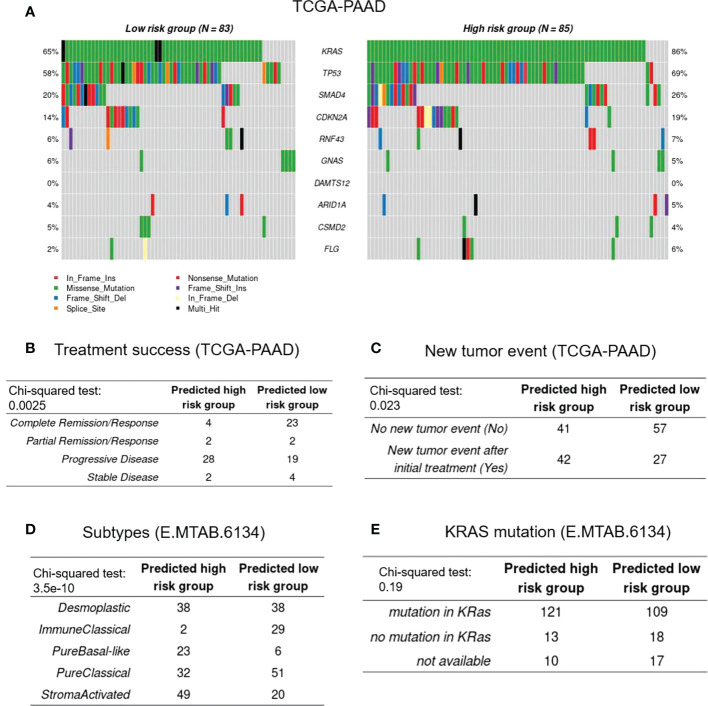
Genetic and clinical association with CAF-based signature. **(A)**, Represents the frequencies of mutated genes among the high- and low-risk groups. **(B-E)**, Represent treatment success, new tumor events, histopathological subtypes, and KRAS mutational status, respectively.

To evaluate the difference in gene mutations between the high- and low-risk cohorts in the TCGA-PAAD database, simple nucleotide variation data were obtained from the GDC database7 and analyzed with the “maftools” package of R. **Figure 5** represents a summary of the gene mutations in the two studied cohorts. As shown in **Figure 5A**, KRAS, TP53, SMAD4, and CDKN2A were the genes with the highest mutation frequencies between the high- and low-risk groups (percentage differences were: 21%, 11%, 6%, and 5%, respectively). Chi square test revealed that the frequency of KRAS mutation in the low-risk group (73 of 85) was significantly higher than that of the high-risk group (54 of 83) (P = 0.00212). Furthermore, in the TCGA-PAAD database, the high-risk groups were shown to have higher numbers of patients with new tumor events, while the low-risk cohorts had better treatment success rates (**Figures 5B, C**). With regard to the clinical and pathological characteristics, data from the E-MTAB-6134 cohort revealed that the number of patients with “pure basal-like” subtype (**Figure 5D**), which has been shown to have the worst prognosis, was higher in the high-risk groups (25), which also showed higher frequencies of KRAS mutations (hypergeometric test; P = 0.052). Moreover, the “stroma-activated” subtype, another dominant subtype identified in our high-risk cohort, has been shown to have lower immune cell infiltration rate and higher fibroblast/endothelial abundance in its TME (26). On the other hand, the low-risk patients showed the “immune classical” subtype, which has been shown to have higher expression levels of CTLA4, sensitizing them to immunotherapy medications (27). Finally, the number of patients with the “pure classical” subtype was higher in the low-risk groups, which has been previously reported to have the best survival rates (28).

### Functional enrichment analysis reveals top molecular pathways in high-risk PDAC patients based on the CAF-based score

**Figure 6:**
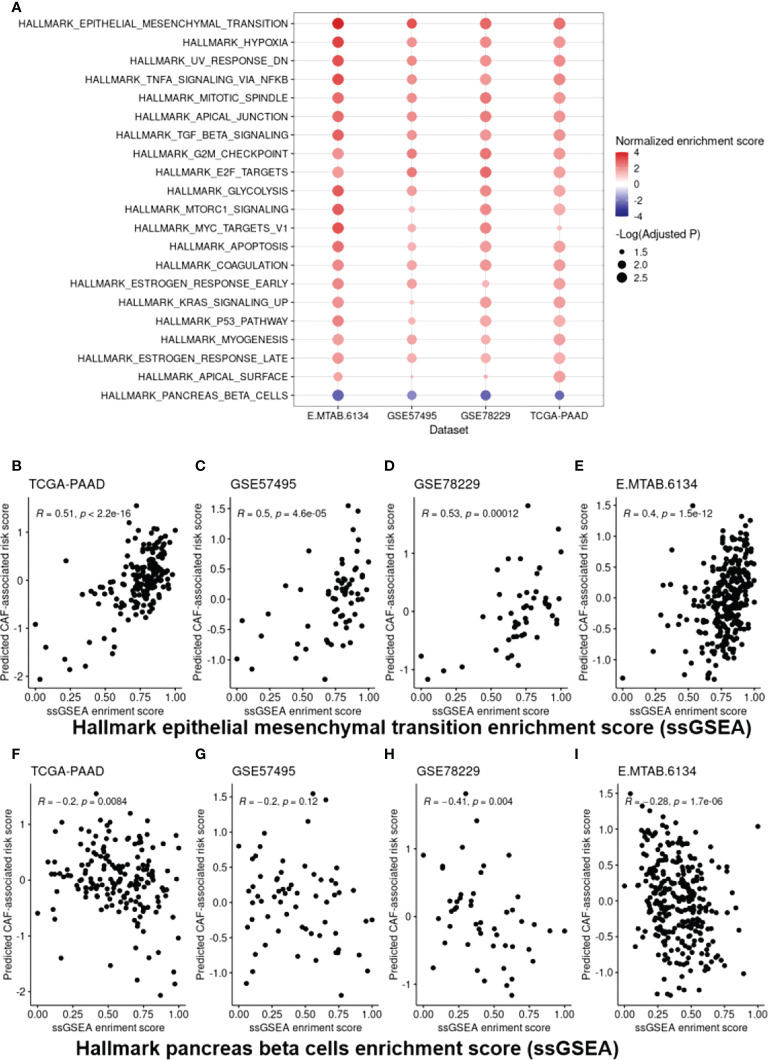
Cancer hallmark gene set analysis. **(A)**, EMT and “pancreas beta cell” gene sets were found to be the top dysregulated hallmark gene sets among all the studied datasets. **(B-I)**, Scatter plots representing the correlation of the CAF-associated risk model with ssGSEA enrichment scores for the EMT **(B-E)** and “pancreas beta cell” gene set **(F-I)** as a further validation step. EMT, Epithelial-Mesenchymal Transition.

Following the identification of the DEGs, Gene Set Enrichment Analysis (GSEA) on hallmarks gene sets from MSigDB was used to determine the top dysregulated biological pathways and/or functional genes in the patients with high CAF-infiltration (**Supplementary Figure 2**). The results revealed significant gene set expression differences between the high- and low-risk groups of patients (**Figure 6A**). Our results showed 21 hallmark gene sets of which “epithelial mesenchymal transition” (EMT) and “pancreas beta cells” had the highest enrichment scores. To further validate the findings, a single sample GSEA (ssGSEA) analysis was also conducted, in which an EMT and beta cell signature enrichment score was allocated to each sample. Our results revealed that the CAF risk score was strongly correlated with the hallmark EMT (**Figures 6B–E**) and pancreas beta cells (**Figures 6F–I**) in TCGA-PAAD, GSE57495, GSE78229, and E-MTAB-6134 datasets. Furthermore, GO and KEGG pathway enrichment analysis were also conducted to enrich the up-regulated genes. Based on the findings, “extracellular matrix organization” and “vasculature development” were among the top enriched pathways in the high-risk patients of the studied cohorts. Taken together, the significant differences in the EMT and the pancreatic beta cell hallmark gene sets between the high- and low-risk groups of patients may at least partially explain the correlation between a high CAF risk score and poor OS.

### The CAF-based signature can predict sensitivity to the most common chemotherapy agents in PDAC treatment

**Figure 7:**
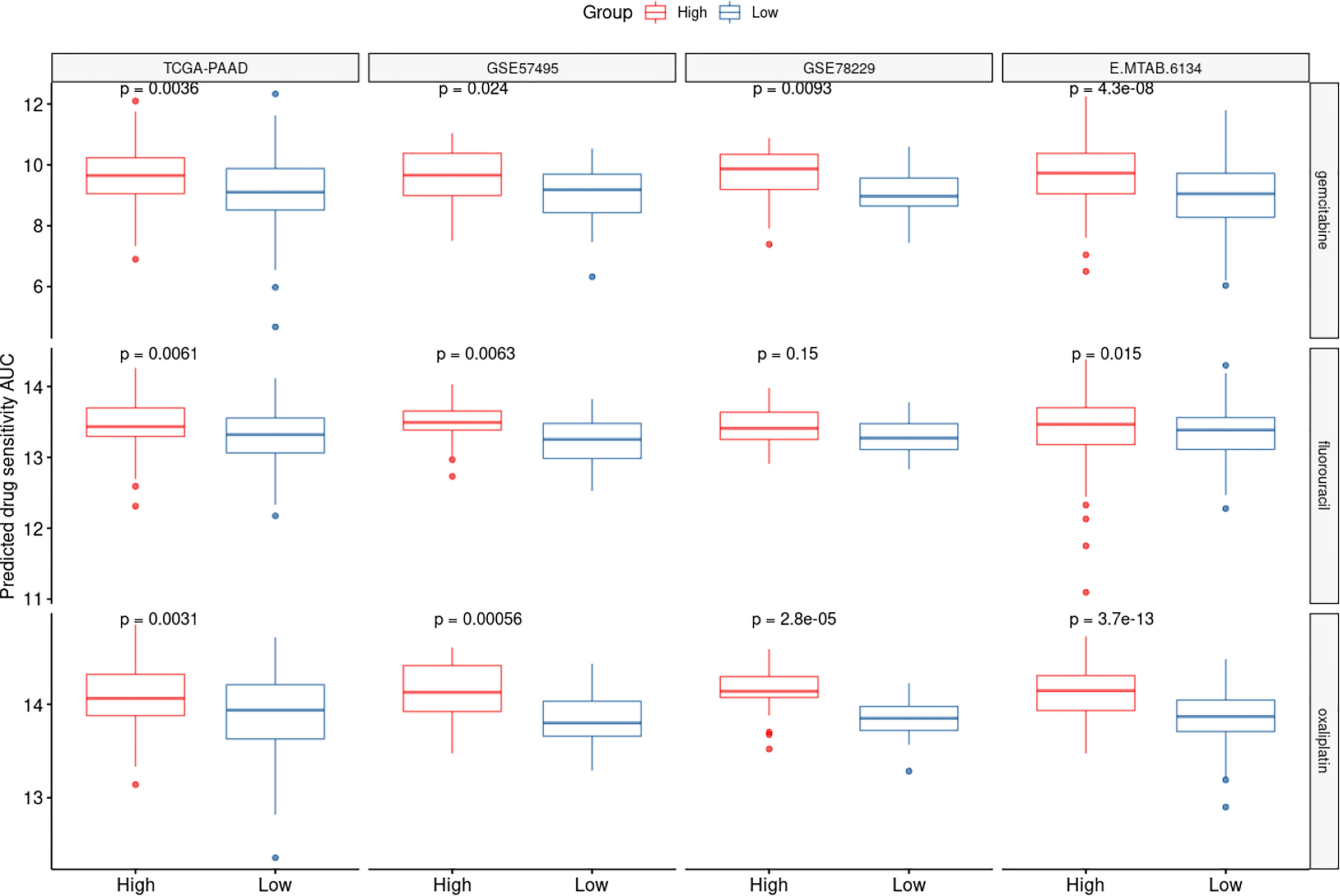
CAF-based signature is associated with chemotherapy response. The drug sensitivity of the high- and low-risk patients to three chemotherapy agents oxaliplatin, 5-fluorouracil and gemcitabine was predicted using AUCs generated by OncoPredict. Lower AUC values represent higher sensitivities.

We utilized the “oncoPredict” tool to estimate sensitivity to frequently-used pancreatic cancer chemotherapy agents to better correlate the CAF-associated gene profiles with clinical practice. Accordingly, drug sensitivity of patients in high- and low-risk groups to multiple chemotherapy agents including gemcitabine, 5-fluorouraci, and oxaliplatin was predicted. Based on the findings, the low-risk groups of patients in all the tested datasets showed higher sensitivity to the aforementioned drugs (**Figure 7**).

## Discussion

Despite the therapeutic advances, the prognosis of pancreatic cancer remains poor (29), highlighting the importance of identifying novel prognostic markers to improve the clinical management of these patients. Nevertheless, the efforts to identify clinically reliable prognostic indicators have not been very successful, necessitating additional studies. Tumor heterogeneity, which is believed to be the primary reason of therapy resistance (30), results from both the tumor cells and their surrounding stromal cells (31). Thus, in addition to the direct study of the tumor cells, clarifying the mechanisms driving this heterogeneity and genomic profiling of the stromal components, could have a significant impact on the prognosis of cancer patients and pave the way to develop novel treatment approaches (32).

In PDAC, fibroblasts play a more pronounced role due to their high abundance which comprise approximately 80% of the tumor bulk and only 20% of the tumor mass involves malignant epithelial cells (33). These fibroblasts mainly originate from pancreatic stellate cells which, under physiologic conditions, function as lipid droplet storages; however, in the cancerous environment, they secrete tumor-stimulating factors and produce ECM, leading to tumor growth, metastasis, and cell survival. Moreover, pancreatic CAFs have been shown to possess different characteristics based on their location within the tumor mass. In this context, Ohlund et al. revealed that multiple subgroups of CAFs with various localizations within the tumor can be identified in PDAC. They specifically mentioned that α-SMA^high^ CAFs can be found in close proximity to the tumor cells, whereas α-SMA^low^ CAFs are localized more distant to these cells, releasing pro-inflammatory cytokines like IL-6 (34). Consequently, due to this high abundance rate and conspicuous heterogeneity of CAFs within the PDAC tumors, their profiling might be helpful in predicting the future behavior of the tumor. Of note, by collecting data from 9356 patients of 32 cancer subtypes from a TCGA pan-cancer cohort, it was shown that the CAF index can outperform other parameters like EMT score in terms of prognosticating survival outcomes (35). Nevertheless, there is currently little evidence available on the prognostic significance of CAF infiltration in patients with PDAC. In order to investigate this hypothesis, CAF infiltration score was estimated in four different datasets from the TCGA, GEO, and ArrayExpress databases. Results from the MCP-counter method revealed that higher CAF infiltration score is associated with OS, indicating a role for CAFs in the progression of PDAC. The constructed CAF-based prognostic risk model also showed that the CAF risk-high groups manifest shorter survival, indicating the fact that CAFs may serve as an independent prognostic marker in PDAC patients. In agreement with these findings, it has been previously shown that higher percentage of stromal infiltration or elevated expression of α-SMA in histological investigations can be a marker of poor clinical outcome in PDAC patients (36). Similarly, with regard to other cancers, Dourado et al. also demonstrated that higher infiltration of CAFs in TME is correlated with worse prognosis in oral squamous cell carcinoma patients (37).

Subsequently, to obtain a CAF-related gene expression signature, genes differentially expressed between the CAF risk-high and -low groups of patients were identified, followed by functional enrichment analysis. Functional enrichment analysis of the 125 identified overlapping DEGs among four different datasets revealed that most of these genes are enriched in “extracellular matrix organization” and “vasculature development” pathways. Supporting these findings, CAFs have been shown to act as a sine qua non of ECM remodeling (38). They produce various ECM proteins, primarily fibrous collagens (types I, III, and V) and fibronectin. Additionally, they alter the ECM by cleaving its constituent proteins with matrix metalloproteinases (MMPs) and crosslinking collagen with enzymes from the lysyl oxidase (LOX) family. This constituent modification of ECM promotes migration and metastasis of cancer cells. Furthermore, CAFs can enhance the “stiffness” of TME, which is linked to chemoresistance and reduced chance of survival in different malignancies (39). Regarding neovascularization, it has documented that both cancer cells and stromal cells affect vasculature development. In fact, CAFs directly enhance tumor angiogenesis through producing pro-angiogenic factors like vascular endothelial growth factor A (VEGFA), fibroblast growth factor 2 (FGF2), CXCL12, and PDGFC (40–42). Beside their direct impacts, modification of ECM proteins like fibronectin, osteopontin, periostin, and collagens by CAFs can also result in vasculature development in solid tumors (43). Additionally, GSEA was carried out to analyze the biological processes and mechanisms through which the CAF-related gene signature can be potentially linked to the prognosis in the CAF risk-high cohorts. Our findings suggested that EMT and hypoxia hallmarks were the most enriched biological processes in the CAF risk-high patients. Additionally, single sample GSEA revealed that the high CAF risk score is correlated with EMT beta cell enrichment score. CAFs have been shown to have the ability to induce EMT by secreting cytokines like IL-6 in the TME (44). In accordance with our results, Ino et al. documented that hypoxia-induced ARG2-expressing CAFs were independent predictors of poor survival in PDAC patients (45). Furthermore, under hypoxic conditions, CAFs can increase motility of PDAC cells through the paracrine signaling of Insulin-like Growth Factor 1 (IGF-1) and its receptor (IGF1R) (46).

We also found that our CAF-based signature is correlated with specific gene mutations as well as certain clinical characteristics of PDAC patients in the studied datasets. Our findings revealed that KRAS mutation is the top-ranked mutation among all other genes in the CAF risk-high patients. Interestingly, through the stromal cells, oncogenic KRAS (KRAS^G12D^) has been shown to control the signaling of tumor cells. In this context, Tape et al. demonstrated that heterotypic fibroblasts are engaged by tumor cell that carry the KRAS^G12D^ mutation, which in turn triggers reciprocal signaling in the malignant PDAC cells (47). Furthermore, tumor recurrence rate was found to be higher in the CAF risk-high groups of patients; while the CAF risk-low patients showed higher treatment success, further supporting the prognostic value of CAFs in PDAC. With regard to the pathological subtypes, the CAF risk-high patients dominantly showed pure basal-like subtype, while immune classical and pure classical were the major subtypes in the CAF risk-low patients. Investigating the prognostic relevance of pancreatic cancer subtypes has revealed that the pure basal-like subtype has the poorest prognosis, with a median OS time of 10.3 months, whereas the pure classical and immune classical subtypes similarly show better a prognosis (median OS values of 43.1 and 37.4 months, respectively) (48).

Of note, recurrence rate among pancreatic cancer patients even after tumor resection at early stages is considerably high (up to 70-80%) (49), which may be at least partially attributed to the inefficacy of current chemotherapies. Accordingly, since we showed that the CAF risk-high patients show higher recurrence rates, we also aimed to investigate whether our CAF-based panel is capable of prognosticating the patients’ response to the most commonly-used chemotherapeutic agents in pancreatic cancer. Our findings revealed that patients with higher risk scores were less sensitive to certain chemotherapy agents including gemcitabine, 5-FU, and oxaliplatin. In support of these findings, Fang et al. demonstrated that pancreatic CAFs induce gemcitabine resistance through the delivery of miR-106b to cancerous cells through secreted exosomes (50). MiR-106b was shown to exert its function by targeting *TP53INP1*, which is a tumor suppressor and autophagy-inducer gene in tumor cells (51). Zhang et al. also reported that higher IL-8 production by PDAC CAFs is correlated with oxaliplatin resistance in these patients. The underlying mechanism of this chemoresistance was attributed to the upregulation of UPK1A-AS1 lncRNA *via* IL-8 signaling, which in turn enhances DNA double-strand break (DSB) repair by strengthening the binding between Ku70 and Ku80 (52). Furthermore, it has been reported that CAFs can induce 5-FU resistance in colorectal cancer through cargo delivery of secreted exosomes (53). However, the role of CAFs in inducing 5-FU resistance has not yet been investigated warranting further investigations.

Finally, our CAF-based prognostic risk model revealed 12 genes which were associated with OS including *ADAMTS12*, *CHST11*, *DCBLD2, FN1, FRMD6, KRT17, LOXL2, MMP14, NRP2, PPFIBP1, TGFB1*, and *VCL.* Among these identified genes *ADAMST12* has been shown to be a poor prognostic marker as well as a role player in invasion and metastasis of PDAC (54). Moreover, Feng et al. also reported that patients with higher expression of *DCBLD2* have lower disease-free survival (DFS) rates (55). Similarly, *MMP14* was also revealed to be a poor prognostic marker for PDAC patients (56). In parallel with our findings in the pathway enrichment analysis, in a pancreatic cancer related complementary EMT model, it was shown that *CHST11*, a modifier of glycosaminoglycan sulfation was significantly upregulated in the EMT model (57). Another study also reported a 5-fold increase in the expression of this gene in pancreatic tumor tissues compared to normal tissues (58). Furthermore, among the identified dysregulated genes, *LOXL2* has well-established associations with cancer invasiveness, metastasis and poor prognosis, which has been also linked to EMT promotion (59–61). Pre-clinical and clinical data on pancreatic cancer has shown that *LOXL2* is correlated with the invasiveness of pancreatic cancer and has the potential to be used as an independent prognostic marker and therapeutic target (59). The other identified genes have been also reported to be involved in the progression of various malignancies through involvement in different major regulatory pathways. For instance, *TGFB1, KRT17, and FRMD6* play critical roles in the TGF-β-related phenotype, mTOR/S6k1, and the Hippo signaling pathways, respectively (62–64). The prognostic values of the other identified genes could be the subject of future investigations.

## Conclusion

Here we report for the first time that higher CAF infiltration is a poor prognosis marker in pancreatic cancer and CAF risk-high patients show lower survival rate. Based on gene enrichment data, “extracellular matrix organization” and “vasculature development” pathways are the top pathways associated the identified CAF-based gene signature. We further showed that high-CAF-risk patients are less sensitive to conventional chemotherapy drugs including gemcitabine, 5-FU, and oxaliplatin and demonstrate higher new tumor events in comparison to the low-CAF-risk groups of PDAC patients. Finally, through prognostic risk model construction, *ADAMTS12*, *CHST11*, *DCBLD2, FN1, FRMD6, KRT17, LOXL2, MMP14, NRP2, PPFIBP1, TGFB1*, and *VCL* were identified as potential prognostic markers in PDAC. These findings emphasize the significance of tumor-extrinsic factors, including the tumor stroma and resident CAFs, in determining the course of tumor progression as well as their clinical significance as indicators of prognosis and therapy responsiveness. However, further clinical studies are warranted to confirm the clinical usefulness of CAF-based gene signatures in PDAC patients.

## Data availability statement

The original contributions presented in the study are included in the article/**Supplementary Material**. Further inquiries can be directed to the corresponding author.

## Author contributions

PL conceived the presented idea and researched the background of the study. JZ, MC and CF prepared the figures and tables. JZ and PL wrote the manuscript. All authors contributed to the article and approved the submitted version.

## Conflict of interest

The authors declare that the research was conducted in the absence of any commercial or financial relationships that could be construed as a potential conflict of interest.

## Publisher’s note

All claims expressed in this article are solely those of the authors and do not necessarily represent those of their affiliated organizations, or those of the publisher, the editors and the reviewers. Any product that may be evaluated in this article, or claim that may be made by its manufacturer, is not guaranteed or endorsed by the publisher.

## Supplementary material

Supplementary Figure 1 Schematic illustration of the study design.

Supplementary Figure 2 Volcano plots for differential expression analysis between high- and low-risk groups across the four datasets.

Supplementary Figure 3 Decision-curve analysis (DCA) plot depicting the net benefit of adopting the risk probability as a continuous predictor for 1-, 3- and 5-year survival.

Supplementary Table 1 Area under the curve (AUC) for the risk predicting the 1-, 3- and 5-year survival.
