# Supplementary figures and images for: A cancer-associated fibroblast gene signature predicts prognosis and therapy response in patients with pancreatic cancer
Source: Front Oncol. 2022 Nov 18;12:1052132. doi: 10.3389/fonc.2022.1052132 (PMC9716208; doi:10.3389/fonc.2022.1052132)

Supplementary Figure 1: Schematic illustration of the study design.

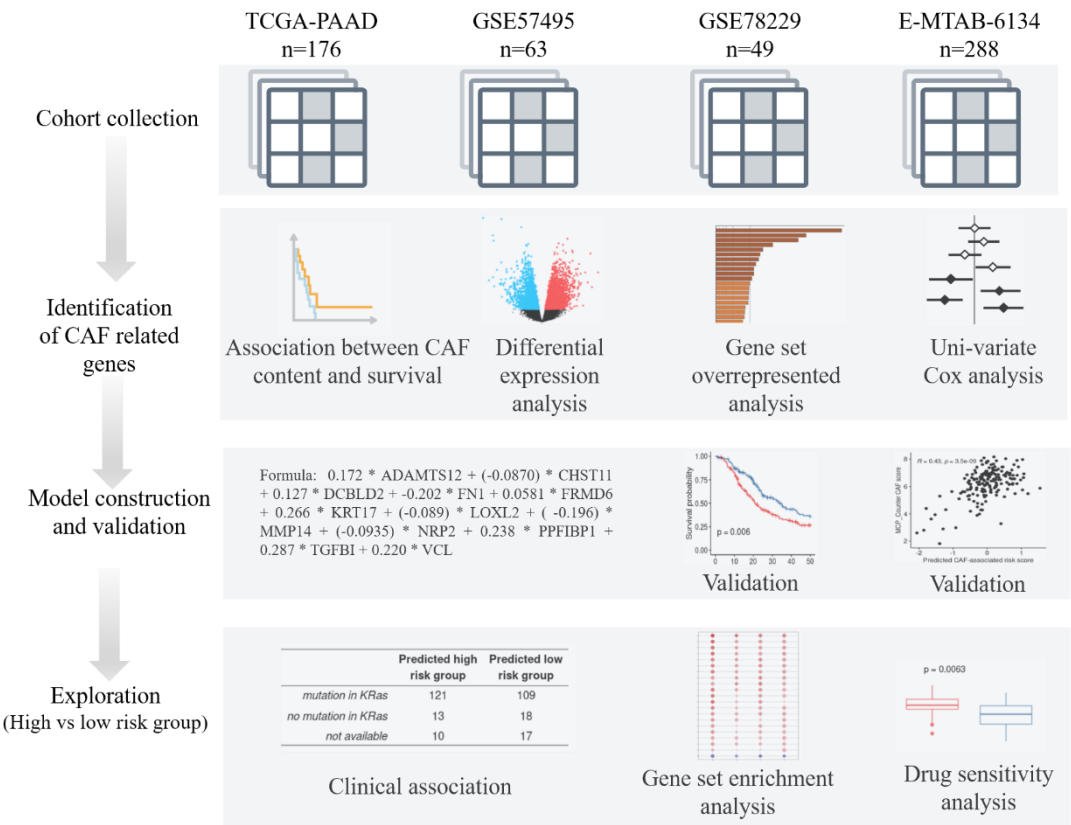

Supplement: Supplementary Figure 1 — Schematic illustration of the study design. [file Image_1.pdf]
