# Supplementary material for: A cancer-associated fibroblast gene signature predicts prognosis and therapy response in patients with pancreatic cancer
Source: Front Oncol. 2022 Nov 18;12:1052132. doi: 10.3389/fonc.2022.1052132 (PMC9716208; doi:10.3389/fonc.2022.1052132)

Supplementary Figure 2: Volcano plots for differential expression analysis between high- and low-risk groups across the four datasets.

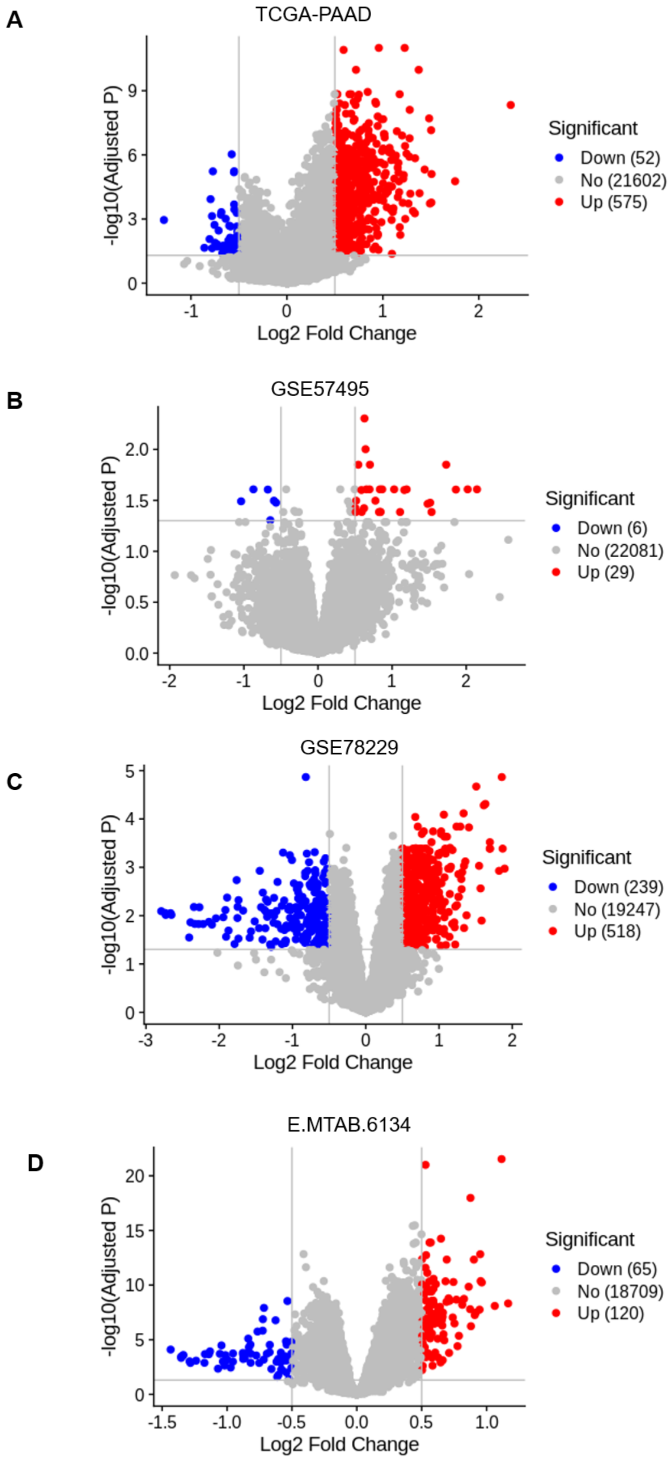

Supplement: Supplementary Figure 2 — Volcano plots for differential expression analysis between high- and low-risk groups across the four datasets. [file Image_2.pdf]
