# Supplementary material for: A cancer-associated fibroblast gene signature predicts prognosis and therapy response in patients with pancreatic cancer
Source: Front Oncol. 2022 Nov 18;12:1052132. doi: 10.3389/fonc.2022.1052132 (PMC9716208; doi:10.3389/fonc.2022.1052132)

Supplementary Figure 3: Decision-curve analysis (DCA) plot depicting the net benefit of adopting the risk probability as a continuous predictor for 1-, 3- and 5-year survival.

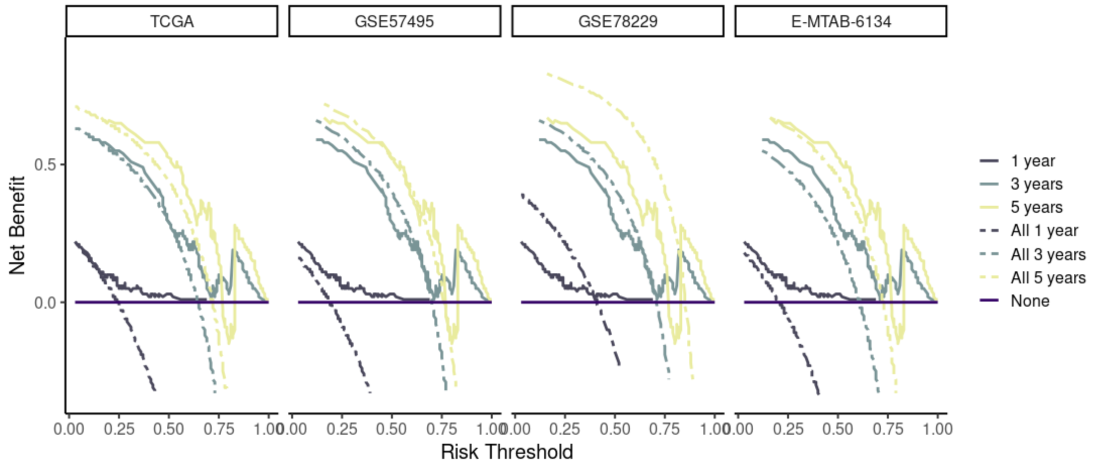

Supplement: Supplementary Figure 3 — Decision-curve analysis (DCA) plot depicting the net benefit of adopting the risk probability as a continuous predictor for 1-, 3- and 5-year survival. [file Image_3.pdf]
