# Supplementary material for: A cancer-associated fibroblast gene signature predicts prognosis and therapy response in patients with pancreatic cancer
Source: Front Oncol. 2022 Nov 18;12:1052132. doi: 10.3389/fonc.2022.1052132 (PMC9716208; doi:10.3389/fonc.2022.1052132)
Supplement: Supplementary Table 1 — Area under the curve (AUC) for the risk predicting the 1-, 3- and 5-year survival. [file Table_1.docx]

**Supplementary Table 1:** Area under the curve (AUC) for the risk predicting the 1-, 3- and 5-year survival.

Supplementary Table 1

|  | 1year | 3 years | 5 years |
| --- | --- | --- | --- |
| TCGA | 0.708 | 0.733 | 0.744 |
| GSE57495 | 0.630 | 0.710 | 0.724 |
| GSE78229 | 0.568 | 0.774 | 0.761 |
| E-MTAB-6134 | 0.654 | 0.589 | 0.617 |
